# SRGN-TGFβ2 regulatory loop confers invasion and metastasis in triple-negative breast cancer

**DOI:** 10.1038/oncsis.2017.53

**Published:** 2017-07-10

**Authors:** Z Zhang, Y Deng, G Zheng, X Jia, Y Xiong, K Luo, Q Qiu, Ni Qiu, J Yin, M Lu, H Liu, Y Gu, Z He

**Affiliations:** 1 Affiliated Cancer Hospital & Institute of Guangzhou Medical University, Guangzhou, Guangdong, China; 2 Guangzhou Institute of Snake Venom Research, School of Pharmaceutical Sciences, Guangzhou Medical University, Guangzhou, Guangdong, China

## Abstract

Patients with triple-negative breast cancers (TNBC) are at a high risk for a recurrent or metastatic disease, and the molecular mechanisms associated with this risk are unclear. Proteoglycan serglycin (SRGN) proteins are involved in tumor metastasis, but their role in TNBC has not yet been elucidated. This study investigates the *SRGN* gene expression and how it regulates TGFβ2 and the downstream signaling of TGFβ2 in TNBC cells and tissues. Our results show that SRGN mRNA and protein expression levels were significantly higher in TNBC cell lines and tumor tissues than that in non-TNBC cells and tissues. We inhibited SRGN expression and protein secretion using shRNA and we observed this inhibited the invasive motility of TNBC cancer cells *in vitro* and metastasis of TNBC cancer cells *in vivo.* SRGN protein treatment increased the expression and secretion of transforming growth factor-β2 (TGFβ2) by activating CD44/CREB1 signaling and promoted epithelial-to-mesenchymal transition in TNBC cells. Moreover, TGFβ2 treatment increased the mRNA and protein expression of the *SRGN* gene by activating Smad3 to target the SRGN relative promoter domain in TNBC cells. Our findings demonstrate that SRGN interacts with TGFβ2 which regulates TNBC metastasis via the autocrine and paracrine routes. SRGN could serve as a potential target for development of agents or therapeutics for the TNBC.

## Introduction

Breast cancer is one of the most common malignancies for women. Although the treatment of breast cancer has greatly improved over the past few decades, there are still around 50 million people worldwide that die from breast cancer every year. The persistence and prevalence of breast cancer may be due to its high genetic heterogeneity, since patients who exhibit the same clinical and histological tumor morphology may have divergent molecular genetic characteristics. These divergences can affect the treatment and prognosis of breast cancer,^1, 2, 3^ however, the molecular events involved in the treatment resistance have not yet been fully elucidated.

Breast cancer is commonly divided into four types: Luminal A (ER^+^, PR^+^, HER2^−^ and low Ki67 expression), Luminal B (ER^+^, PR^+^, HER2^−/+^, high Ki67 or any level expression), HER2 overexpression (ER^−^, PR^−^, HER2^+^), or basal-like. Approximately 80% of basal-like breast cancer are ER^−^, PR^−^ and HER2^−^, which are called TNBC− (triple-negative breast cancer). TNBC is a very heterogeneous group of cancers that is aggressive, has a high risk of relapse, has poor prognosis, and often occurs in women under 50 years of age. Due to lack of endocrine therapy and HER2 targeted therapy, the main clinical treatments of TNBC rely on chemotherapy which causes therapeutic resistance.^4^ Therefore, it is important to determine the molecular mechanism underpinning metastasis and recurrence of TNBC.

Serglycin (SRGN) is a low molecular weight glycoprotein involving in breast cancer metastasis. SRGN can be secreted from cells and integrated into the extracellular matrix. SRGN consists of a core protein with 158 amino acids that is attached with various mucopolysaccharides (GAGs). The core protein forms three function regions: signal peptide (amino acid residues 1–27), N-terminal (amino acid residues 28–76), and C-terminal (amino acids 77–158). The C-terminal of SRGN contains multiple serine and glycine repeat regions which bind to the GAGs.^5, 6^ Studies have shown that SRGN is mainly expressed in blood cells, endothelial cells, tumor cells, and embryonic stem cells. SRGN has an important role in the storage and secretion of a variety of proteases, chemokines and cytokine.^7, 8^

The effect of SRGN in cancer was first found as a marker to distinguish myeloid leukemia from lymphoid leukemia.^9^ In multiple myeloma, high expression of SRGN can inhibit the complement activity, which can help tumor cells to escape from immune surveillance.^10^ Elevated SRGN expression can be used as a prognostic indicator of liver cancer.^11^ Recent studies have shown that SRGN can induce an epithelial–mesenchymal transition (EMT) in nasopharyngeal carcinoma cells and aggressive breast cancer cells, promoting tumor cell invasion and metastasis.^12, 13^ Lung metastasis of breast cancer is significantly inhibited in SRGN-deficient mice.^14^ It is suggested that SRGN may be highly associated with breast cancer metastasis, possibly through regulating EMT activation. In this study, we investigated the expression and biological functions of *SRGN* gene in TNBC breast cancer cells and observed which signal pathways were affected.

## Results

### SRGN mRNA and protein expression increases in breast cancer cells and tissues

**Figure 1:**
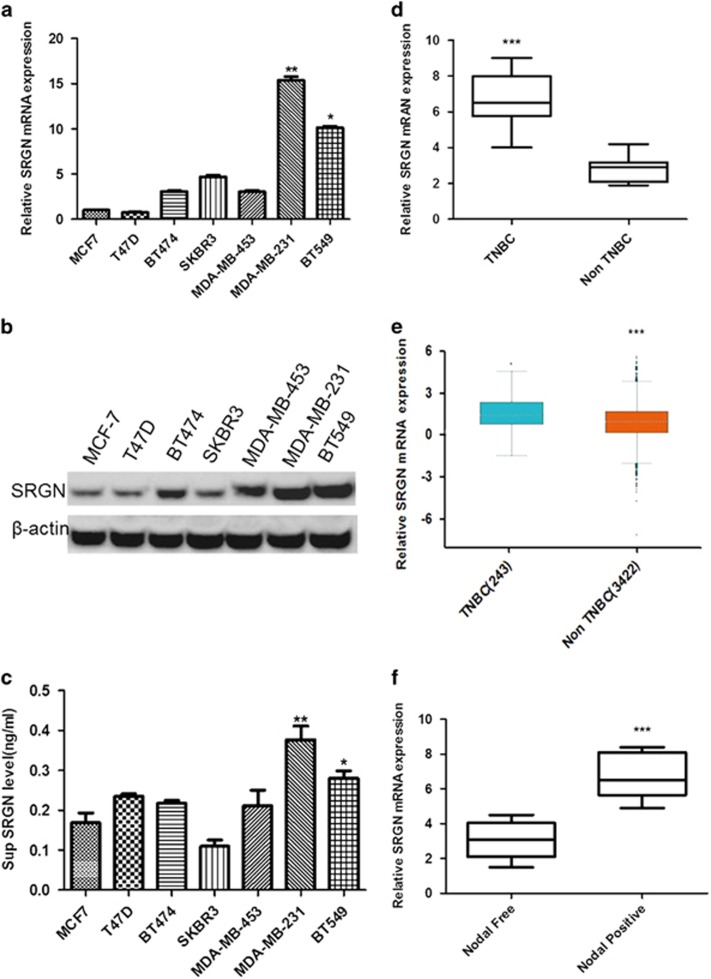
SRGN expression in TNBC. (**a**) Real-time PCR of *SRGN* mRNA expression in breast cancer cell lines. *P<0.05, **P<0.01 vs MCF cells. *N*=3. (**b**) Western blot of SRGN protein expression in breast cancer cell lines. (**c**) ELISA assay of SRGN concentration in the supernatant (Sup) of cultured breast cancer cells. *P<0.05, **P<0.01 vs MCF cells. *N*=3. (**d**) Real-time PCR of *SRGN* mRNA expression in triple-negative breast cancer (TNBC) and non-TNBC tumor tissues. ***P<0.001 vs non-TNBC. (**e**) Breast Cancer Gene-Expression Miner v4 analyzes *SRGN* mRNA expression in basal-like and TNBC and non basal-like & non-TNBC tissues. ***P<0.001 vs non basal-like and non-TNBC. (**f**) Real-time PCR of *SRGN* mRNA expression in breast cancer tumors with positive lymph node metastases (Nodal positive) (*n*=135) and negative lymph node metastases (Nodal free) (*n*=142). ***P<0.001 vs node free.

SRGN has been demonstrated to induce proliferation, migration and invasion in breast cancer cells,^13^ but its expression in human breast cancer tissues and differential expression in different cell lines has not been reported. We therefore monitored *SRGN* mRNA expression using real-time PCR and measured protein expression by western blot in six breast cancer (BC) cell lines. *SRGN* mRNA (Figure 1a) and protein (Figure 1b) expression levels were significantly higher in the TNBC cells (MDA-MB-231 and BT549 cell), than other subtypes of BC cells (MCF7, T47D, BT474 and SKBR3 cells) (*P*<0.05, *P*<0.01 vs MCF cells). We also measured the SRGN protein level in the supernatant of cultured cells by ELISA and found that the supernatant SRGN level in TNBC was significantly higher than other types of BC cells (*P*<0.05, *P*<0.01 vs MCF cells, Figure 1c). We further measured mRNA expression of the *SRGN* gene using RT–PCR in tissues from 106 cases of TNBC and 320 cases of other BC types (non-TNBC) and we found that TNBC tumors contained significantly higher SRGN mRNA levels compared with other BC types (*P*<0.001, Figure 1d). We searched our observed mRNA expression pattern of the human *SRGN* gene against the Breast Cancer Gene-Expression Miner v4.0 database (http://bcgenex.centregauducheau.fr) and again found that mRNA expression of *SRGN* gene was significantly higher in basal-like and TNBC tumors than that in non basal-like and non-TNBC tumors (*P*<0.001, Figure 1e).

We wanted to understand the association between *SRGN* gene expression and breast cancer progression and metastasis, and collected a panel of breast cancer tissue samples and divided them into a positive lymph node metastases group (Node positive) (*n*=135) and a negative lymph node metastases group (Node negative) (*n*=142). The SRGN mRNA levels were significantly higher in the ‘Nodal positive’ group than that in the ‘Nodal negative’ group (*P*<0.001, Figure 1f). These results suggest that SRGN gene is highly expressed TNBC cells and tissues compared with other BC types and associated with the metastatic phenotype of BC.

### SRGN promotes TNBC cells migration, invasion and metastasis

**Figure 2:**
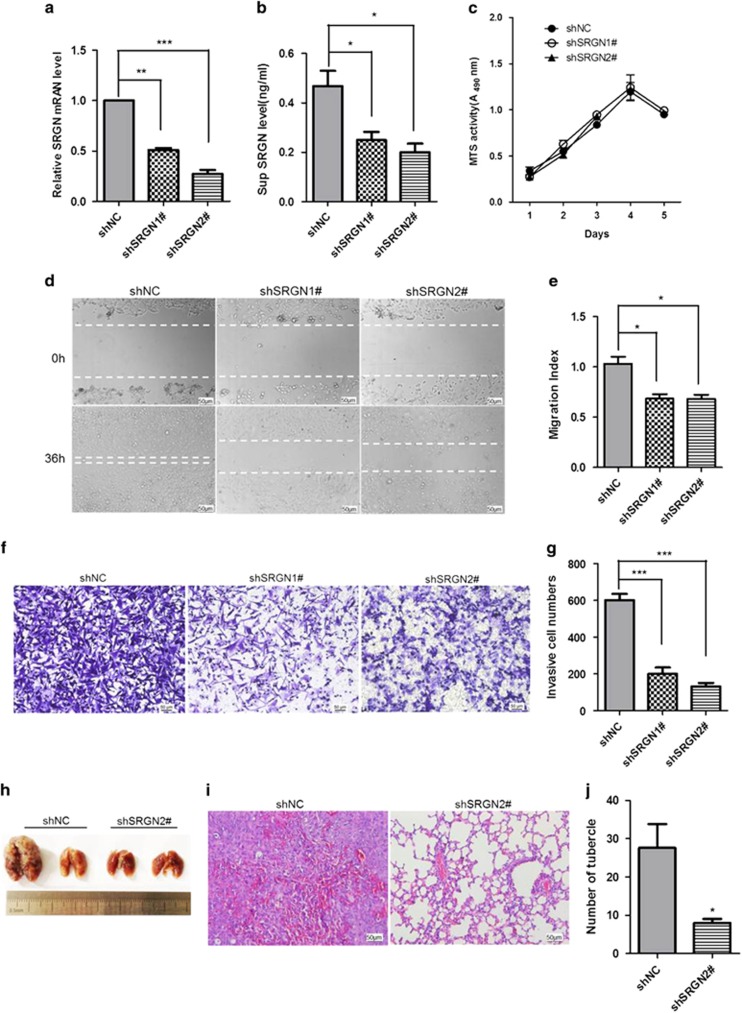
SRGN promotes metastasis of breast cancer cells. (**a**) Real-time PCR of SRGN mRNA expression in MDA-MB-231 stable cells. SRGN mRNA expression was significantly decreased in MDA-MB-231/shSRGN1# (shSRGN1#) and MDA-MB-231/shSRGN2# (shSRGN2#) stable cells compared with control stable MDA-MB-231 cells (shNC). **P<0.01. *N*=3. (**b**) ELISA assay of SRGN concentration in the supernatant of cultured MDA-MB-231 stable cells. *P<0.05 between two groups. *N*=3. (**c**) MTS detection of proliferation ability of MDA-MB-231 stable cells. (**d**) Representative *in vitro* scratch assay. (**e**) The migration index of *in vitro* scratch assay in **c**. *P<0.05 between two groups. *N*=3. (**f**) Representative cell invasion test in Transwell assay. (**g**) The cell invasion index of invasion test in Transwell. ***P<0.001 between two groups. *N*=3. (**h**) Representative lung showing metastasis. (**i**) Representative HE staining of lung tissues. (**j**) The number of tubercles in the lung between control MDA-MB-231 stable cell (shNC) and MDA-MB-231/shSRGN2# stable cell (shSRGN2#) inoculated mice. *P<0.05 vs shNC. Scale bar, 50 μm.

Although one study investigated the biological functions of SRGN in breast cancer cells by overexpression of serglycin,^13^ the affection of silencing *SRGN* gene expression on the proliferation, migration, and invasion of BC cells has not been reported. To determine the biological significance of SRGN, we knocked down *SRGN* gene expression by transfecting MDA-MB-231 cells with two shRNAs (shSRGN1# and shSRGN2#) and established stable cell lines with these shRNAs (MDA-MB-231/shSRGN1# and MDA-MB-231/shSRGN2#) using lentiviral vectors. The *SRGN* mRNA expression in cells and SRGN protein concentration in the supernatant of cultured cells were measured by real-time PCR and ELISA, respectively. The SRGN mRNA expression in the cells (Figure 2a) and SRGN protein concentration (Figure 2b) in the supernatant of MDA-MB-231/shSRGN1 (shSRGN1#) and MDA-MB-231/shSRGN2 (shSRGN2#) stable cells were significantly decreased compared with MDA-MB-231 cell stably expressing non-target sequence (shNC) (mRNA: *P*<0.01, *P*<0.001, Protein: *P*<0.05). MTS analysis showed that silencing of *SRGN* gene expression had no obvious effect on the viability of MDA-MB-231 cells *in vitro* (*P*>0.05) (Figure 2c). However, in the xenograft tumor models, tumors inoculated with MDA-MB-231/shSRGN2# cells (shSRGN2#) grew significantly slower than tumors inoculated with MDA-MB-231/shNC (shNC) (*P*<0.01) (Supplementary Figure 1A). Tumors were smaller and in MDA-MB-231/hSRGN2# than in MDA-MB-231/shNC (*P*<0.05) (Supplementary Figures 1B–D). The *in vitro* scratch assay showed that MDA-MB-231/shSRGN1# (shSRGN1#) and MDA-MB-231/shSRGN2# (shSRGN2#) stable cells migrated significantly slower than MDA-MB-231/shNC stable cells (Figure 2d and e). The Transwell invasion assay showed that significantly less MDA-MB-231/shSRGN1# (shSRGN1#) and MDA-MB-231/shSRGN2# (shSRGN2#) stable cells invaded to the lower chamber compared with the MDA-MB-231/shNC stable cells (Figure 2f and g). To further evaluate the effect of SRGN on the metastasis of MDA-MB-231 cells *in vivo*, MDA-MB-231/shSRGN2# and MDA-MB-231/shNC stable cells were injected into mice via tail vein and then lung metastases were analyzed 5 weeks later. Significantly less nodules were observed in the lung of mice injected MDA-MB-231/shSRGN2# stable cells than mice injected MDA-MB-231/shNC stable cells (*P*<0.05, Figures 2h–j). These findings suggest that silencing of SRGN gene expression significantly inhibited cell migration and invasion of MDA-MB-231 cells *in vitro* and tumor growth and metastasis of xenograft MDA-MB-231 tumors *in vivo*.

We also investigated the biological effects of *SRGN* gene expression in one non-TNBC cell line MCF7 with low endogenous SRGN expression. The SRGN mRNA (Supplementary Figure 2A) and protein (Supplementary Figure 2B) expression in MCF7 cells stably overexpressing SRGN gene (MCF7-SRGN) was significantly increased compared with that in control MCF7 sable cells (MCF7-NC). We also observed that the protein level (Supplementary Figure 2C) in the supernatant of cultured MCF7-SRGN stable cells was significantly increased compared with control (*P*<0.001). The *in vitro* scratch assay showed that MCF7-SRGN stable cells (MCF7-SRGN) migrated significantly faster than MCF7-NC control stable cells (Supplementary Figure 2D). The Transwell invasion assay showed that significantly more MCF7-SRGN stable cells (MCF7-SRGN) invaded to the lower chamber compared with the MCF7-NC stable cells (*P*<0.01, Supplementary Figure 2E,F). These results suggest that SRGN overexpression promotes breast cancer cells invasion and metastasis.

### SRGN increases TGFβ2 levels and activated EMT

**Figure 3:**
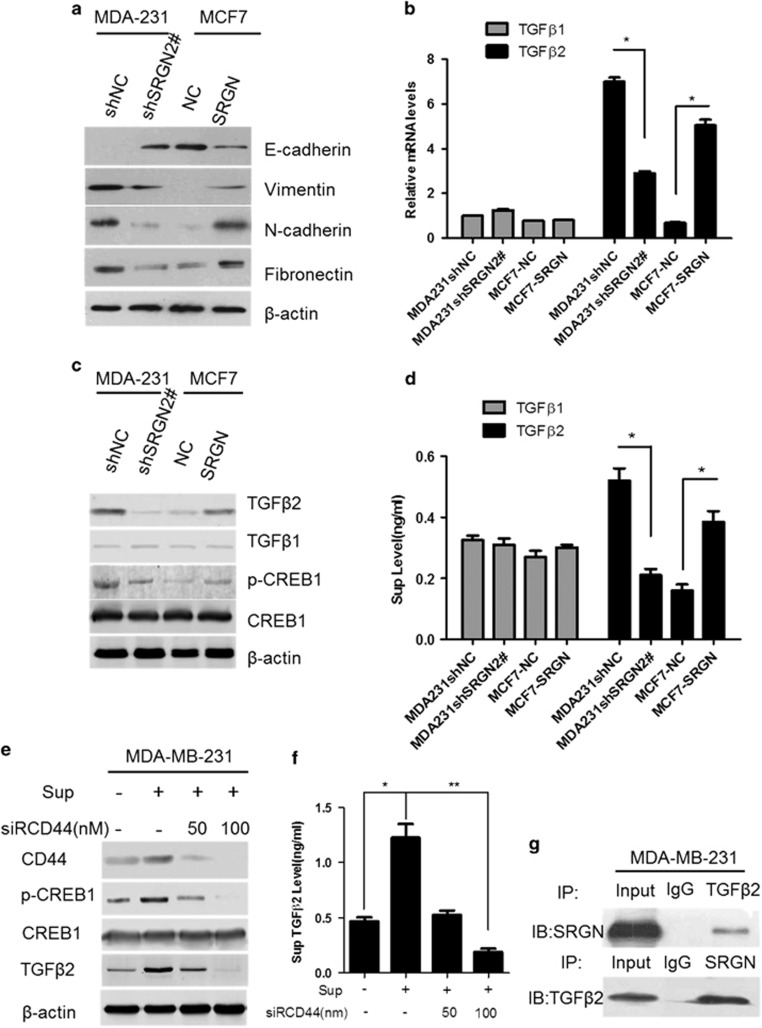
SRGN induces TGFβ2 expression and activates EMT formation. (**a**) Representative western blots of EMT-related markers in MDA-MB-231/shSRGN2#, MDA-MB-231/shNC, MCF7/NC and MCF7/SRGN stable cells. (**b**) Real-time PCR of TGFβ1 and TGFβ2 expression after interference and overexpression of SRGN. *P<0.05 between two groups. *N*=3. (**c**) Representative western blots of TGFβ1, TGFβ2 and phospho-CREB1 protein expression in MDA-MB-231/shSRGN2#, MDA-MB-231/shNC, MCF7/NC and MCF7/SRGN stable cells. (**d**) ELISA assay of TGFβ1 and TGFβ2 concentration in the supernatant of cultured MDA-MB-231/shSRGN2#, MDA-MB-231/shNC, MCF7/NC and MCF7/SRGN stable cells. *P<0.05 between two groups. *N*=3. (**e**) Representative western blots of CD44, TGFβ2, CREB1 and phospho-CREB1 in MDA-MB-231 cells treated with supernatant (Sup) of cultured MCF7/SRGN and siRNA of CD44 gene (siRCD44). (**f**) ELISA assay of TGFβ2 concentration in the supernatant of cultured MDA-MB-231 cells treated with cell supernatant from cultured MCF7/SRGN cells and siRCD44. *P<0.05, **P<0.01 between two groups. *N*=3. (**g**) Co-immunoprecipitation of SRGN with TGFβ2 in MDA-MB-231 cells.

A previous study demonstrated that SRGN can induce an epithelial–mesenchymal transition (EMT) in nasopharyngeal carcinoma cells^12^ and the cytokine transforming growth factor β (TGFβ) is known to trigger the EMT process.^15^ In addition, the E-cadherin to N-cadherin switch is a biomarker for EMT. The increased vimentin expression is associated with a migratory phenotype, and up-regulated fibronectin expression is observed in EMT.^16^ We wanted to know whether SRGN activates EMT in BC cells through regulating *TGFβ2* gene expression. Western blots showed that E-cadherin was significantly increased in MDA-MB-231-shSRGN2# cells stably expressing shRNA2# of *SRGN* gene (shSRGN2#), but was significantly decreased in MCF7 cells overexpressing *SRGN* gene compared with their control groups, respectively (Figure 3a). In contrast, the protein expression of vimentin, N-cadherin, and fibronectin was significantly decreased in MDA-MB-231-shSRGN2# stable cells (shSRGN2#), but was significantly increased in MCF7-SRGN stable cells overexpressing *SRGN* gene (SRGN) compared with their own control groups (Figure 3a). These findings suggest that SRGN activates EMT.

Real-time PCR showed that *TGFβ2* mRNA was significantly decreased in MDA-MB-231-shSRGN2# stable cells (MDA231 shSRGN2#) compared with MDA-MB-231-shNC stable cells (MDA231 shNC) (*P*<0.05), but significantly increased in MCF7-SRGN stable cells compared with MCF7-NC stable cells (MCF7-NC) (*P*<0.05) (Figure 3b). No significant differences in *TGFβ1* mRNA expression were observed between MDA231 shSRGN2# and MDA231 shNC, and between MCF7-SRGN and MCF7-NC stable cells (*P*>0.05) (Figure 3b). Western blots showed that TGFβ2 and pCREB1 protein expression was decreased in MDA-MB-231-shSRGN2# stable cells (shSRGN2#) compared with that in MDA-MB-231-shNC stable cells (shNC), but increased in MCF7-SRGN stable cells compared with that in MCF7-shNC stable cells (shNC) (Figure 3c). No obvious differences in TGFβ1 and CREB1 protein expression were observed between MDA231 shSRGN2# and MDA231 shNC, and between MCF7-SRGN and MCF7-NC stable cells (*P*>0.05) (Figure 3c). The concentration of TGFβ1 and TGFβ2 protein in the supernatant of cultured cells was measured by ELISA (Figure 3d). TGFβ2, but not TGFβ1 concentration was significantly decreased in the supernatant of cultured MDA-MB-231-shSRGN2# stable cells (MDA231 shSRGN2#) compared with that in the supernatant of cultured MDA-MB-231-shNC stable cells (MDA231 shNC) (*P*<0.05), but significantly increased in the supernatant of cultured MCF7-SRGN stable cells compared with that in the supernatant of cultured MCF7-NC stable cells (*P*<0.05) (Figure 3d). These findings suggest that SRGN positively regulated TGFβ2, but not TGFβ1 protein expression and increased CREB1 phosphorylation, but not CERB1 protein expression.

TGFβ2 can promote the expression of CREB1 in tumor cells^23^ and CD44 is a classic marker of breast cancer stem cells.^17^ We wanted to know whether SRGN can regulate CD44 TGFβ2 protein expression and CREB1 phosphorylation. We therefore tested CERB1 protein expression and phosphorylation in MDA-MB-231 cells to observe the effects of exogenous SRGN on CD44 and TGFβ2 protein expression. The exogenouse SRGN was the supernatant collected from the cultured MCF7-SRGN stable cells. The CD44 gene expression was silenced by a siRNA of the CD44 gene (siRCD44). MDA-MB-231 cells were treated with the supernatant (sup) collected from the cultured MCF7-SRGN stable cells, with or without 50 and 100 nm siRCD44 for 48 h. Western blots showed that the supernatant from cultured MCF7-SRGN stable cells significantly increased CD44 and TGFβ2 protein expression, CREB1 protein phosphorylation, but not CREB1 protein expression in MDA-MB-231 cells (Figure 3e). In contrast, 50 nm and 100 nm siRCD44 can block the effects of the supernatant from cultured MCF7-SRGN stable cells on CD44 and TGFβ2 protein expression, and CREB1 protein phosphorylation and even significantly decreased these proteins’ levels compared with that in the untreated cells (−/−) (Figure 3e). ELISA assay showed that the supernatant from cultured MCF7-SRGN stable cells significantly increased, but siRCD44 significantly decreased TGFβ2 protein concentration in the supernatant of cultured MDA-MB-231 cells (*P*<0.05, *P*<0.01) (Figure 3f). Co-immunoprecipitation analysis showed that SRGN directly bound to TGFβ2 in MDA-MB-231 cells (Figure 3g). These results indicate that exogenous SRGN can stimulate CD44 and TGFβ2 protein expression, and CREB1 phosphorylation in MDA-MD-231 cells, and TGFβ2 protein secretion from MDA-MD-231 cells. SRGN may interact with TGFβ2 in MDA-MD-231 cells.

### TGFβ2 regulates SRGN expression

**Figure 4:**
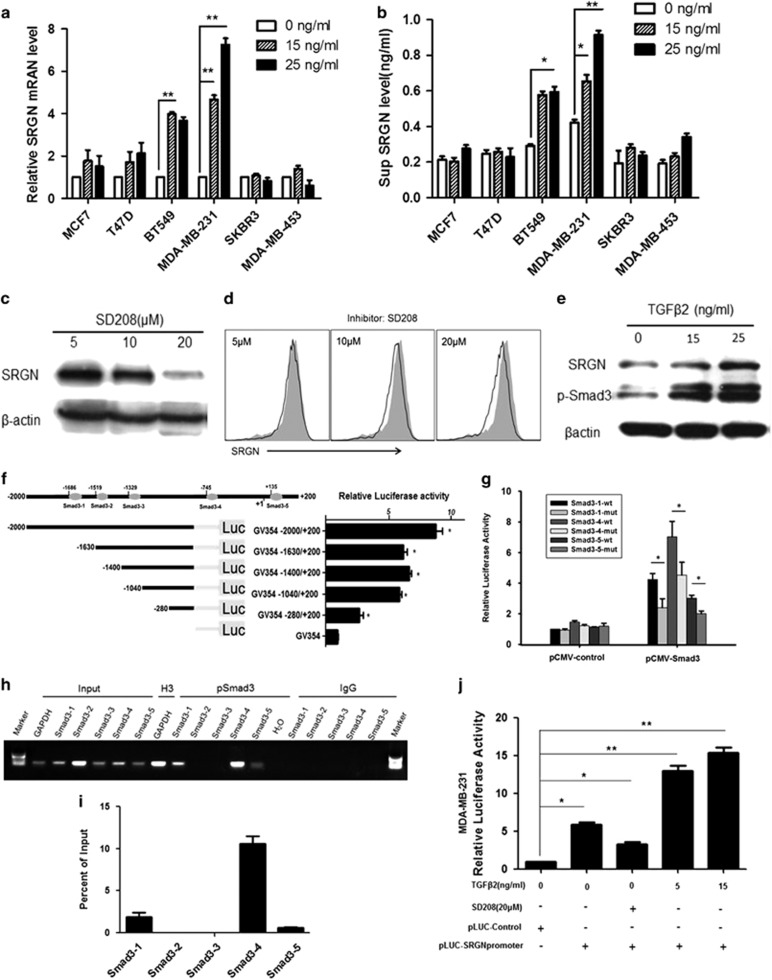
TGFβ2 regulates SRGN expression through positive feedback. (**a**) Real-time PCR of SRGN mRNA expression in cells treated with different TGFβ2. **P<0.01 between two groups. *N*=3. (**b**) ELISA assay of SRGN concentration in the supernatant of cells treated with different concentration of TGFβ2. *P<0.05, **P<0.01 between two groups. *N*=3. (**c**) Representative western blots of SRGN protein expression in MDA-MB-231 cells treated with different concentration of TGFβ receptor inhibitor SD208. (**d**) Flow cytometry assay of SRGN protein expression on the cell membrane in MDA-MB-231 cells treated with different concentration of TGFβ receptor inhibitor SD208. (**e**) Representative western blots of SRGN and phospho-Smad3 protein expression in MDA-MB-231 cells treated with different concentration of TGFβ2. (**f**) Bioinformatics prediction of the SMAD3 binding sites in the SRGN promoter region (−2000 to +200) and relative luciferase (LUC) activity in 293 T cells after transforming with reporter vector containing different length of promoter region. *P<0.05 vs GV345, *N*=3. (**g**) Luciferase reporter gene activity in 293 T cells transformed with reporter vector containing wild-type (wt) SRGN promoter sequence and reporter vector containing SRGN promoter sequence with mutated (mut) Smad3 1, 4, 5-binding sites. *P<0.05 between two groups. *N*=3. (**h**) CHIP-qPCR to verify SMAD3 binding sites in MDA-MB-231 cells. Input: no antibody control; H3: chromatin protein H3; pSmad3: pSmad3 antibody; IgG: IgG a control. (**i**) Quantitative analysis of the effective that CHIP-qPCR validated the possible binding sites of the SMAD3 in the SRGN promoter region. (**j**) Relative luciferase activity in MDA-MB-231 cells treated with TGFβ2, TGFβ receptor inhibitor, transformation of control, and SRGN promotor reporter vector. *P<0.05, **P<0.01 between two groups. *N*=3.

We want to know whether TGFβ2 has a feedback regulation on SRGN and treated MDA-MB-231, BT549, MCF7, T47D, BT474, and SKBR3 cells with 0, 15 and 25 ng/ml of TGFβ2 for 48 h. Real-time PCR showed that TGFβ2 treatment significantly increased SRGN mRNA expression in TNBC cell lines BT549 and MDA-MB-231 compared with other cell types (Figure 4a). ELISA assay showed that TGFβ2 significantly increased SRGN concentration in the supernatant of cultured BT549 and MDA-MB-231 cells compared with other cell types (Figure 4b). TGFβ receptor inhibitor SD208 was used to further confirm the regulatory role of TGFβ2 signaling on SRGN protein. SD208 treatment dose-dependently down-regulate SRGN protein levels in whole cells (Figure 4c) and cell membrane (Figure 4d) in MDA-MB-231 cells. Similar to TGFβ2, TGFβ1 also significantly increased the mRNA and protein expression of *SRGN* gene as well as secretion of SRGN protein in TNBC cell lines BT549 and MDA-MB-231 (Supplementary Figure 3). These findings suggest that both TGFβ1 and TGFβ2 can regulate SRGN gene expression in TNBC cells.

We want to know how the TGFbeta regulates *SRGN* gene expression in TNBC cells and performed a bioinformatic assay, which predicted that SMAD3 may have five binding sites in the *SRGN* promoter. We therefore tested which binding site has a key role in regulating *SRGN* gene expression. Western blots showed that TGFβ2-stimulated SRGN protein expression correlated with the increases in Smad3 protein phosphorylation in MDA-MB-231 cells (Figure 4e). The bioinformatic analysis predicted five potential SMAD2/3 binding sites (GTCT/AGAC) in the SRGN promoter region (−2000 to +200). To validate it, five different 5'deletion sequences containing different Smad3 binding sites were cloned into the promoter- luciferase reporter vector pGL4. The constructs were transfected into 293 T cells. Results from this deletion analysis showed that the Smad3 binding sites 1, 4, 5 were essential for the promoter activity upon treatment with 10 ng/ml TGFβ2 (Figure 4f). The pLuc vector containing the SRGN promoter with mutated 1, 4 or 5-binding site of Smad3 (Samd3-1-mut, Samd3-4-mut, Samd3-5-mut) or wild-type Samd3 binding site (Samd3-1-wt, Samd3-4-wt, Samd3-5-wt) was co-transfected with the pRL-TK Renilla luciferase vector with or without the pCMV-SMAD3 vector (pCMV-Samd3) or control vector (pCMV-control) into 293 T cells. The luciferase activity assay showed that pCMV-SMAD3 vector transfection significantly increased luciferase activity in cells transfected with pLuc vector containing wild-type Samd3 binding site compared with transfection with pCMV-control vector (*P*<0.05) (Figure 4g). Also, in cells transfected with pCMV-Smad3 vector, the luciferase activity was significantly decreased when co-transfected with pLuc vector containing wild-type Samd3 binding site compared with co-transfected with pLuc vector containing mutated Samd3 binding site (Figure 4g). To further verify the binding sites of SMAD2/3 in SRGN promoter region, we performed CHIP-PCR, which also confirmed that SMAD2/3 could bind to site 1,4,5 in SRGN promoter region (Figure 4h and i). The pLuc vector containing the SRGN promoter (pLuc-SRGN promoter) and pLuc control vector (pLuc Control) were transfected into MDA-MB-231 cells which expresses endogenously TGFβ2. Result showed that pLuc-SRGN promoter transfection significantly increased luciferase activity in MDA-MB-231 cells compare to the pLuc Control transfection (*P*<0.05), but 20-μM of SD208 inhibitor partially reversed the effect of pLuc-SRGN promoter transfection (*P*<0.05) (Figure 4j). Addition of exogenous TGFβ2 further dose-dependently increased the luciferase activity in MDA-MB-231 cells (Figure 4j). These results suggest the existence of Smad3 binding site in SRGN promoter and TGFβ2 can regulate SRGN protein expression through increasing Smad3 activation.

### SRGN and TGFβ2 expression are positively associated in TNBC tissues and serum

**Figure 5:**
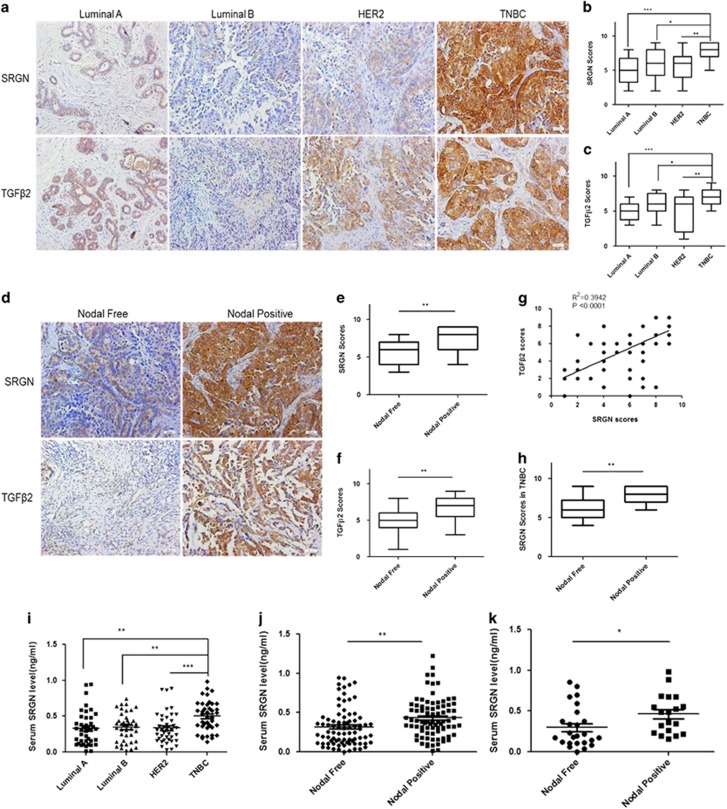
SRGN and TGFβ2 expression in tumor tissues of breast cancer patients. (**a**) Representative immunohistochemical staining of SRGN and TGFβ2 protein in tumor tissues with different types of breast cancer. (**b**) Histological scores of SRGN staining in tumor tissues. *P<0.05, **P<0.01, ***P<0.001 between two groups. (**c**) Histological scores of TGFβ2 staining in tumor tissues. *P<0.05, **P<0.01, ***P<0.001 between two groups. (**d**) Representative immunohistochemical staining of SRGN and TGFβ2 protein in tumor tissues in patients with and without lymph node metastasis. (**e**) Histological scores of SRGN staining in breast cancer patients with and without lymph node metastasis. **P<0.01 between two groups. (**f**) Histological score of TGFβ2 staining in breast cancer patients with and without lymph node metastasis. **P<0.01 between two groups. (**g**) Correlation analysis of the expression of SRGN and TGFβ2 protein in TNBC tissues. (**h**) Histological scores of SRGN staining in TNBC patients with and without lymph node metastasis. **P<0.01 between two groups. (**i**) ELISA assay of serum SRGN concentration in patients with different types of breast cancer. **P<0.01, ***P<0.001 between two groups. (**j**) ELISA assay of serum SRGN concentration in breast cancer patients with and without lymph node metastasis. **P<0.01 between two groups. (**k**) ELISA assay of serum SRGN concentration in TNBC patients with and without lymph node metastasis. *P<0.05 between two groups. Scale bar, 50 μm.

To further validate the association between SRGN and TGFβ2 protein expression in breast cancer patients, we detected the SRGN and TGFβ2 protein expression in 320 non-TNBC tumor tissues (110 Luminal A, 108 Luminal B, and 102 HER2) and 106 TNBC tumor tissues by immunohistochemical staining. SRGN (Figures 5a and b) and TGFβ2 (Figures 5a and c) protein expression in TNBC tumor tissues was significantly higher than that in non-TNBC tumor tissues [Luminal A (*P*<0.001), Luminal B (*P*<0.05), and HER2 (*P*<0.01)]. The expression of SRGN and TGFβ2 protein in breast cancer tumors with (Nodal Positive, *n*=135) and without (Nodal Free, *n*=142) lymph node metastases was also detected by immunohistochemical staining (Figure 5d). Significantly higher scores of positive SRGN (Figure 5e) and TGFβ2 (Figure 5f) staining was observed in breast cancer tumors with lymph node metastases (Nodal Positive) compared with breast cancer tumors without lymph node metastases (Nodal Free) (*P*<0.01). The scores of SRGN staining positively correlated with the scores of TGFβ2 staining in TNBC tissues (Figure 5g), and was significantly higher in TNBC tissues with lymph node metastasis (Nodal Positive) than in TNBC tissues without lymph node metastasis (Nodal Free) (Figure 5h). We measured the SRGN concentration in the serum of patients by ELISA. The concentration of serum SRGN was significantly higher in patients with TNBC (TNBC, Figure 5i), in breast cancer patients with lymph node metastasis (Nodal Positive, Figure 5j), and in TNBC patients with lymph node metastasis (Nodal Positive, Figure 5k) than that in patients without TNBC [Luminal A (*P*<0.01), Luminal B (*P*<0.01), and HER2 (*P*<0.001)] (Figure 5i), in BC patients without lymph node metastasis (Nodal Free) (*P*<0.01) (Figure 5j), and TNBC patients without lymph node metastasis (Nodal Free) (*P*<0.05) (Figure 5k), respectively. These data suggest that SRGN up-regulation correlates with TGFβ2 in triple-negative breast cancers which may lead to eventually metastasis.

## Discussion

**Figure 6:**
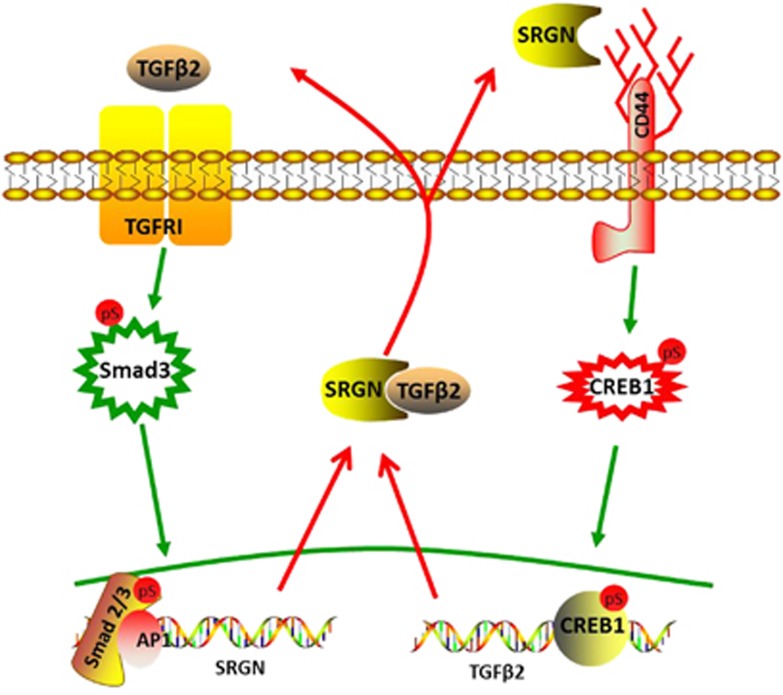
Interaction of SRGN with TGFβ2. In TNBC, SRGN enhances TGFβ2 expression and secretion of matured TGFβ2 which subsequently activates EMT through binding to CD44 and phosphorylating CREB1. At the same time, TGFβ2 regulates SRGN transcriptional expression through binding to TGFβ receptor, and subsequently activates the downstream SMAD2/3, and positively regulating SRGN expression.

TNBC is difficult to treat due to its high metastasis rate, its susceptibility to relapse, and the lack of information regarding its specific targets. This study found that glycoprotein SRGN is highly expressed in the TNBC tumor cells. SRGN enhances TGFβ2 expression and matured TGFβ2 extracellular secretion and subsequently activates EMT through binding CD44 and phosphorylation of CREB1. At the same time, TGFβ2 is highly expressed in TNBC and regulates SRGN transcriptional expression by binding to TGFβ receptor, which subsequently activates the downstream SMAD2/3 and positively regulates SRGN expression (Figure 6). Therefore, activation of the SRGN-TGFβ2 loop in TNBC is essential for maintaining the high metastatic potential of TNBC.

SRGN is a small molecule glycoprotein mainly distributing in the cell and cell membrane, but it can also be secreted and integrated into the extracellular matrix. Studies have shown that SRGN promotes cell migration by binding to its receptor in CD44 in blood cells.^18^ CD44 is thought to be a classic marker of breast cancer stem cells and has an important role in tumor stem cell adhesion, invasion, metastasis, apoptosis resistance, chemoresistance, and EMT formation.^17^ For example, the binding of CD44 to its classical ligand, hyaluronic acid (HA), activates protein kinase C, which in turn activates transcription factor SMAD2/3, and subsequently has an important role in EMT formation.^19, 20^

Here, we observe that SRGN can promote the expression of TGFβ2 in TNBC cells through binding to its CD44 ligand, which subsequently activates CREB1. TGFβ2 is known activator of EMT which can explain the effect of SRGN in the formation of EMT. In addition, SRGN can also mediate the maturation and secretion of MMP9 precursors.^21^ This suggests that SRGN may also promote metastasis and EMT though regulating MMP9 and other metal matrix protease molecules. Moreover, TGFβ family members have multiple disulfide bonds, and one of them is mainly involved in the intermolecular linkage.^22^ This explains our finding that immunoprecipitation results in SRGN directly bound to TGFβ2 through the disulfide bond, which can promote the partial secretion and maturation of TGFβ2.

A previous study showed that the 5′-UTR of SRGN contains multiple (−80) ETS and (−70) CRE sites which mainly regulate the expression of SRGN, though different stimulus conditions can regulate different sites.^23^ In the present study, we found that TGFβ2 regulates the expression of SRGN mainly through the (−745) SMAD3 site. Some studies have shown that TGFβ2 can promote the expression of CREB1 in tumor cells through activating the TGFβ receptor and form a self-activating loop.^24, 25^ In addition to our findings on the SRGN-TGFβ2 loop, we find that TGFβ2, but not TGFβ1, may have a crucial role in TNBC. Therefore, we postulate that TGFβ2 may be an ideal target for future targeting therapy. In addition, our study demonstrates that SRGN does not affect cell growth *in vitro*, but decreases the ability of tumor formation *in vivo* (Supplementary Figure 1), suggesting that tumor microenvironment has a great impact on SRGN tumorigenesis.

Previous studies demonstrated that SRGN is mainly involved in immune response, such as promoting the TNFα secretion from macrophages, and inhibiting classical and non-classical complement activation pathways.^8^ This implies that SRGN may have an important role in tumor immune escape, which may diminish the effect of antibody-dependent complement killing of the current tumor targeted therapy. In addition, TGFβ2 also functions as an important immunosuppressive factor in the formation of tumor immunosuppressive microenvironment.^26^ In this study, the SRGN-TGFβ2 loop may be an important mechanism for TNBC tumor cells to escape immune surveillance. This is worthy of further study.

In summary, our finding of a SRGN-TGFβ2-positive feedback loop highlights a new target for the therapy of refractory and high recurrence of TNBC breast cancer.

## Materials and methods

### Reagents

Antibodies for E-cadherin, N-cadherin, vimentin, fibronectin, CREB1, pCREB1, pSMAD2/3, and CD44 were purchased from Cell Signaling Technology (Beverly, MA, USA). Antibodies for TGFβ1, TGFβ2, and β-actin were obtained from Santa Cruz Biotechnology (Santa Cruz, CA, USA). The antibody for SRGN and anti-Rabbit FITC second antibody were purchased from Abcam (Cambridge, MA, USA). The recombinant human TGFβ1 and TGFβ2 protein was obtained from R&D Systems (Minneapolis, MN, USA). The TGFβ receptor I kinase inhibitor SD208 was from Selleckchem (Houston, TX, USA) and MTS (3-(4,5-dimethyl-2-yl)-5-(3-carboxymethoxyphenyl)-2-(4-sulfophenyl)-2H-tetrazolium, inner salt) was from Promega (Madison, WI, USA). siRNA of CD44 (siRCD44) was purchased from RiboBio (Guangzhou, China) and transformed using Lipofectamine 2000 (Invitrogen, Carlsbad, CA, USA).

### Specimens

The tumor tissues of 426 breast cancers were collected from January 2008 to 2014 at the Affiliated Tumor Hospital of Guangzhou Medical University. The serum of 163 breast cancer patients was collected between 2013 and 2015. The information on the immunohistological staining of ER, PR, HER2, Ki67, and clinical classifications of breast cancer patients were recorded. The tissue and serum samples were divided into Luminal A, Luminal B, HER2 positive, and TNBC group. Fresh tumor tissues were collected from 135 breast cancer patients with lymph node metastasis and 142 patients without metastasis from 2012 to 2015 to detect RNA. This study was approved by the Ethics Committee of the Affiliated Cancer Hospital, Guangzhou Medical University and informed consent was waived.

### Cell culture and proliferation assay

Breast cancer cell lines MCF7, T47D, BT474, SKBR3, MDA-MB-231 and BT549, and HEK293T human embryonic kidney cell line were obtained from the American Type Culture Collection (ATCC) and cultured in Dulbecco's Modified Eagle's (DMEM) medium (Gibco, Carlsbad, CA, USA) containing 10% fetal bovine serum (Gibco) at 37 °C, 5% CO_2_ (Thermo, Waltham, MA, USA). Cell growth curves were plotted by using the cellular viability values assessed by the MTS method using CellTiter 96 Aqueous One Solution Cell Proliferation Assay System (Promega).

### Establishment of stable cell lines

The plenshSRGN-GFP lentiviral vector overexpressing shRNA1 (5′-gaactacttccaggtgaatcc-3′) and shRNA2 (5′-ggaacaggattaccaactagt-3′) to silence *SRGN* gene expression, pEZ-SRGN lentiviral vector overexpressing *SRGN* gene, and control lentiviral vectors (NC) were purchased from Genecopoeia (Guangzhou, China). MDA-MB-231 cells at 70% confluence were transfected with plenshSRGN-GFP lentiviral vector and control vector using BLOCK-iT Lentiviral Pol II miR RNAi system (Invitrogen) by following the user manual. Twenty-four hours after transfection, fresh medium containing 2 μg/ml of puromycin (Invitrogen) was added to cells and refreshed every three days for three weeks. Single colonies were then selected, identified, and continuously cultured. This procedure produced two SRGN shRNA stable expression cell lines and a control stable cell line, which we call MDA-MB-231/shSRGN1#, MDA-MB-231/shSRGN2#, and MDA-MB-231/shNC, respectively. MCF7 cells were transfected with pEZ-SRGN lentiviral vector to overexpress *SRGN* gene and transfected with pEZ-NC lentiviral to express the blank vector as control as described above. Stable cells were selected using 1.5 μg/ml of puromycin. The produced MCF7 cells stably expressing SRGN were named MCF-SRGN. The control MCF7 stable cell line was called MCF7-NC.

### Cell lysis and western blot

Cultured cells were washed twice before lysis with ice-cold 1 × PBS (phosphate-buffered saline). Cells were then lysed using RIPA (radioimmunoprecipitation assay) buffer supplemented with proteases and phosphatases inhibitors (Sigma-Aldrich, Sigma, St Louis, MO, USA). Protein concentration in lysates was determined by a Pierce BCA protein assay kit (Thermo Scientific, Waltham, MA, USA). Equal amount of protein between samples was separated by electrophorese on SDS–PAGE gel and transferred to polyvinylidene difluoride membranes (Merck Millipore, USA). Membranes were incubated with 5% non-fat milk in TBS (Tris-buffered saline) buffer for an hour at room temperature and then incubated with primary antibodies overnight at 4 °C. The membranes were washed three times with 1 × TBS buffer before incubation with LICOR 680 nm or 800 nm fluorescent secondary antibodies for one hour. After washing with 1 × TBS buffer, the membranes were scanned on a LICOR Odyssey system. The acquired images were analyzed with Image Studio Version 4.0 software according to manufacturer’s instructions.

### Real-time PCR

Total RNA was isolated from breast cancer cells and tumor tissues using TRIzol regents by following the manufacturer’s instructions (Invitrogen). cDNA was generated using SuperScriptIII cDNA kit (Invitrogen). The real-time PCR was performed using ABI7500 Fast Real-Time PCR System. SRGN was amplified using forward primer: 5′-TCCAACAAGATCCCCCGTCT-3′ and reverse primer: 5′-TTCCGTTAGGAAGCCACTCC-3′). The beta-actin was amplified using forward primer: 5′-AGCACAGAGCCTCGCCTTT-3′ and reverse primer: 5′-ATCATCATCCATGGTGAGCTGG-3′ as an internal control. Data were analyzed using 2^−^^ΔΔCT^ method.

### ELISA (enzyme-linked immunosorbent assay)

The SRGN concentration in the serum of breast cancer patients and the supernatant of serum-free cultured cells was measured for 48 h using SRGN ELISA Kit (CUSABIO, China). The concentrations of TGFβ1 and TGFβ2 in the supernatants of cell culture were measured using Human TGF-β1 Quantikine ELISA Kit (PDB110B) and Human TGF-β2 Quantikine ELISA Kit (PDB250) (R&D Systems), respectively according to the manufacturer's instructions.

### *In vitro* migration and invasion assays

An *in vitro* scratch assay and Transwell assays were used to evaluate the migration and invasion abilities of the tumor cells, respectively. Briefly, cells were grown at monolayer in 12-well plates (2 × 10^5^) for overnight. An artificial scratch was created in cells, and cell debris was removed by washing with 1 × PBS. Cell migration was photographed and the width of the wound was measured. Cell invasion was evaluated by a Transwell system. The polycarbonate filters (8-μm pore size) were pre-coated with Matrigel Matrix (BD Biosciences, Franklin Lakes, NJ, USA), and reconstituted at 37 °C for 30 min. Cells (1 × 10^5^) were suspended in 150 μl of serum-free RPMI 1640 medium and added into the upper chamber, while 600 μl of complete medium was added to the lower chamber. The cells that migrated through the matrigel and adhered onto the lower chamber after 24 h of incubation were fixed with 4% paraformaldehyde for 20 min, and then stained with Mayer’s hematoxylin (Sigma-Aldrich, USA), and counted under microscope (five fields per chamber).

### Immunoprecipitation assays

MDA-MB-231 cells were washed twice with ice-cold 1 × PBS, and then lysed with RIPA buffer supplemented with protease inhibitors. Protein concentrations were determined as described above. One milligram of lysates was incubated with protein G-agarose and anti-SRGN or TGFβ2 antibody for 4 h at 4 °C. Beads were washed 3 times with immunoprecipitation (IP) buffer (150 mm NaCl, 25 mm Tris-HCl pH 7.5, 0.1% Nonidet P-40), and proteins were eluted with 1.5 × SDS–PAGE sample buffer. Samples were analyzed by Western blot with anti-SRGN or anti-TGFβ2 antibody.

### Flow cytometry

Cultured MDA-MB-231 cells were treated with or without different concentrations of SD208 inhibitor for 48 h. The suspended cells (1 × 10^6^) were stained with SRGN (1:50) antibody. After 30 min incubation on ice, the cells were washed with staining buffer (BD Biosciences) and then incubated with FITC-labeled second antibody for 30 min on ice in the dark. After washing, the cells were resuspended in staining buffer. Data were acquired using a BD CantonII Flow Cytometer (BD Bioscience) and analyzed using FlowJo software (Ashland, OR, USA).

### Luciferase report assay

To determine whether SMAD3 regulates the promoter activity of SRGN, a region of 2 kb (kilobases) upstream of the first exon of SRGN was cloned into the pLuc-reporter vector upstream of the luciferase gene (Genechem, Shanghai, China). Bioinformatic assay predicted that SMAD3 may have five binding sites (−2000 to +200, −1630 to +200, −1400 to +200, −1040 to +200, −280 to +200) in the SRGN promoter. Therefore, five pGL4 reporter vectors were constructed to contain 1 to 5 proposed SMAD3 binding sites. At the same time, three mutation vectors were constructed using the TaKaRa MutanBEST Kit according to the user manual. The mutation primers included: Site 1: forward primer: 5′-GCCggtaccGGGGGAGCGGTAGGGATAGAC-3′ and reverse primer: 5′-GCCagatctGGCTTAATGCACGTGCCCC-3′ Site 4: forward primer: 5′-GCCggtaccCTACTAAAAATACAAAATTAGTCCAGCGC-3′ and reverse primer: 5′-GCCagatctGACGTGTCACCATGTTGGCCA-3′ and Site 5: forward primer: 5′-GCCggtaccTCAGGAGTCTTGTTCCCCAGC-3′ and reverse primer: 5′-GCCagatctTACCTTGAACTGAGGATTCCAGAAC-3′. Briefly, cells were seeded in 96-well plates and co-transfected with the pLuc vector and the pRL-TK Renilla luciferase vector with or without the pCMV-SMAD3 vector using Lipofectamine 2000 (Invitrogen). After 48 h, luciferase activity was determined using a Dual-Luciferase Reporter Assay System (Promega). The firefly luciferase activity was calculated as the mean±s.d. after normalization with the Renilla luciferase activity.

### CHIP-PCR

The ChIP assay was performed using the EZ-CHIP chromatin immunoprecipitation kit (Merck Millipore, Germany). Briefly, 1% formaldehyde was added to the cultured cells to cross-link the chromatin proteins to DNA. After incubation for 10 min at room temperature, the cells were washed and then scraped off with ice-cold PBS containing protease inhibitors. Cells were pelleted, resuspended, and subjected to sonication to get about 200–1000 base pairs of DNA. After removing cell debris, the samples were diluted 10-fold in ChIP dilution buffer containing protease inhibitors. Five-μg of anti-H3 antibody (positive control was provided with the kit), or anti-pSMAD2/3 antibody (Cell Signaling Technology) were added to the chromatin solution and incubated overnight at 4 °C with rotation. Protein G-agarose was then added and incubated at 4 °C for two hours. The protein/DNA complexes were eluted with ChIP elution buffer. DNA was released form protein/DNA complexes by incubation with 5M NaCl at 65 °C for 4 h. The DNA was purified and 50 μl of DNA was obtained for each treatment. 0.2 μl of DNA from each group was used as a template for PCR. Primers for the SRGN promoter containing putative SMAD3 binding sites included forward primer: 5′-AAACTCCTCCCTCCTATCAA-3′, reverse primer: 5′-AGCCTATCATACATCCTTGC-3′ for site 1; forward primer: 5′-TGTATTTATTGTATAACTTT-3′ and reverse: 5′-TGCCTGTAGTCCCAGCTACT-3′ for site 2; forward primer: 5′-CTCGCCACCA CGCCCGGCTA-3′ and reverse primer: 5′-GGCTCCATATTAAAGTTATA-3′ for site 3; forward primer: 5′-GTTTGCTGGGCACGGTGGCT-3′ and reverse primer: 5′-CCTGAGTAGCTGGGATTACA-3′ for site 4; forward primer: 5′-AAGAAGTTGGCGTGCAGCTG-3′ and reverse primer: 5′-ATGGACCACAGGGCTTACAG-3′ for site 5. The primers amplify human GAPDH gene used forward primer: 5′-TACTAGCGGTTTTACGGGCG-3′ and reverse prime: 5′-TCGAACAGGAGGAGCAGAGAGCGA-3′. The PCR conditions were as follows: one cycle at 95 °C, 5 min; 32 cycles at 95 °C, 20 s, 60 °C, 30 s, and 72 °C, 30 s; and then one cycle at 72 °C for 7 min. PCR samples were electrophoresed on 2% agarose gels and stained with ethidium bromide. In addition, real-time PCR was carried out according to standard protocols using an ABI7500 with SYBR Green detection (Applied Biosystems).

### Histological analysis

Tissues were fixed in 10% neutral buffered formalin for 48 h and then transferred to 70% ethanol before embedding. Tissues were sectioned at 4-μm, mounted on DakoFlex slides (Dako, Denmark), and stained with haematoxylin & eosin.

All reagents used for immunohistochemistry were obtained from Beyotime Institute of Biotechnology (Beijing, China). Sections (4-μm) were deparaffinized in xylene. Endogenous peroxidase was blocked with 3% hydrogen peroxide in deionized water for 20 min. Antigen was retrieved in citrate buffer (10 mm, pH 6.0) at 95 °C for 30 min. Sections were stained with SRGN (1:100) and TGFβ2 (1:100) antibody for 1 h at 37 °C, followed by biotinylated secondary antibody for 30 min, reaction with horseradish peroxidase for 30 min, and visualization with hydrogen peroxide-activated diamino benzidine. After washing with 1 × PBS, sections were counter-stained with hematoxylin, dehydrated using ethanol, cleared with xylene, and mounted in mounting medium. Sections treated without primary antibodies were used as negative controls. The investigators who performed histological analysis were blinded to the group allocation during the experiment and when assessing the outcome.

### Animal experiments

The immunocompetent female BALB/c mice (10–11 weeks old) were purchased from Guangdong Animal Center (China) and randomly grouped. The mice were first injected with 1 × 10^5^ of MDA-MB-231/shSRGN or MDA-MB-231/shNC stable cells via tail vein (*N*=8). After 5 weeks, the rats were killed to observe pulmonary metastases. In addition, 1 × 10^6^ of MDA-MB-231/shSRGN or MDA-MB-231/shNC stable cells were injected subcutaneously into the right posterior of the mice to observe tumor growth. Tumor volume was calculated using *V*=*a*^2^ × *b*/2 (*a*=width, *b*=length). Tumor growth and metastasis were observed by two investigators blinding to the experimental design through recording GFP signals using a small animal live-body imager (Bruker *in vivo* Fx pro, USA). Briefly, mice were anesthetized (inhalation) and placed on the imaging platform. The fluorescent signal was detected with a filter of 520 nm and an excitation of 480 nm. Tumor size and location was observed. All animal experiments were approved by the Experimental Animal Ethics Committee of Guangzhou Medical University.

### Statistics

Statistical analyses were performed using a GraphPad Prism version 5.0 software (GraphPad Software Inc., La Jolla, CA, USA). A two-tailed Fisher’s exact test was used to determine if the frequency distribution were statistically significant. Comparison of treatments was performed using one-way analysis of variance with Newman–Keuls post-test or a paired two-way Student’s *t*-test. Differences were considered statistically significant at values of *P*<0.05.
