# Supplementary figures and images for: SRGN-TGFβ2 regulatory loop confers invasion and metastasis in triple-negative breast cancer
Source: Oncogenesis. 2017 Jul 10;6(7):e360–. doi: 10.1038/oncsis.2017.53 (PMC5541705; doi:10.1038/oncsis.2017.53)

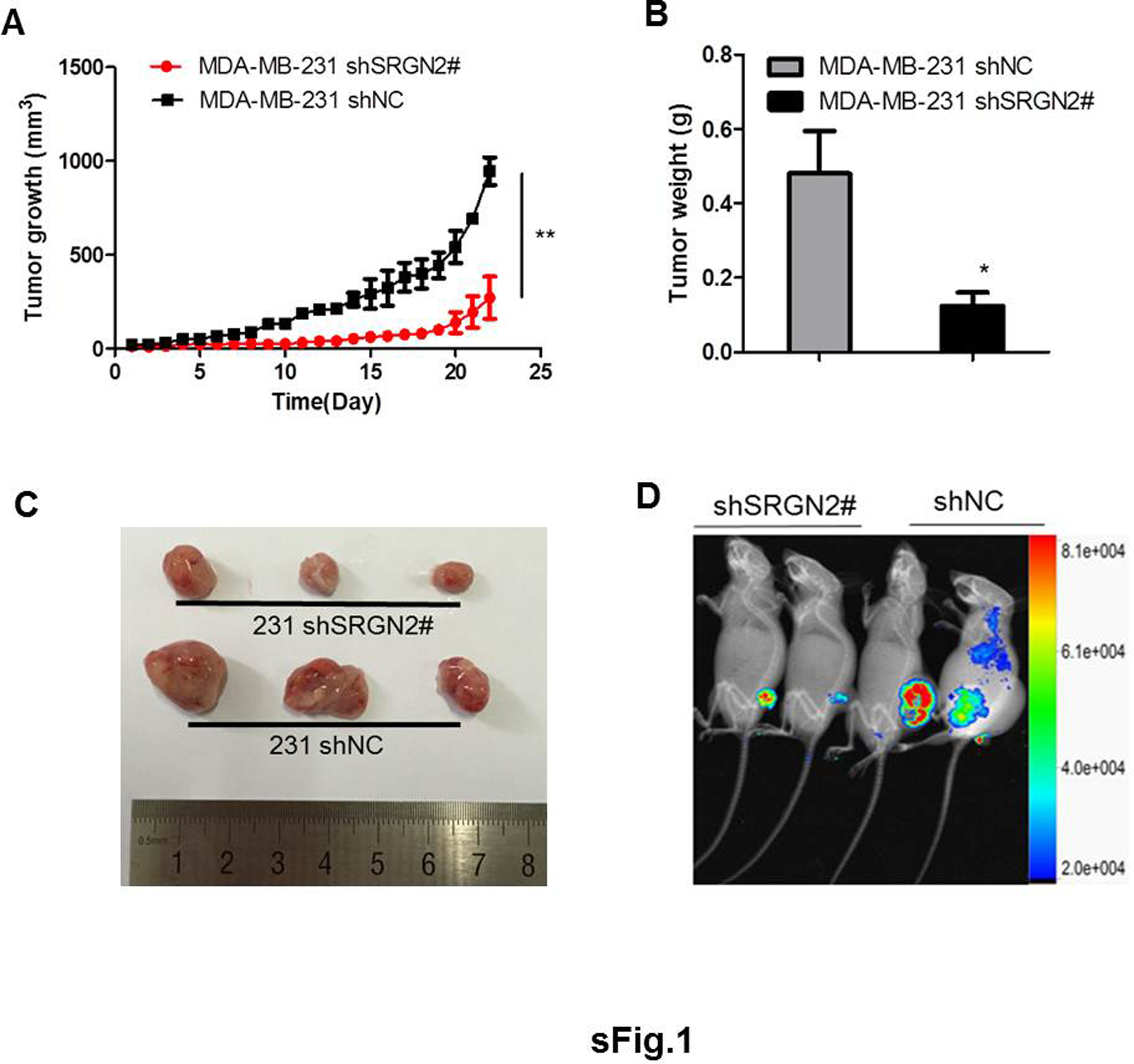

Supplement: Supplementary Figure 1 [file oncsis201753x1.tif]

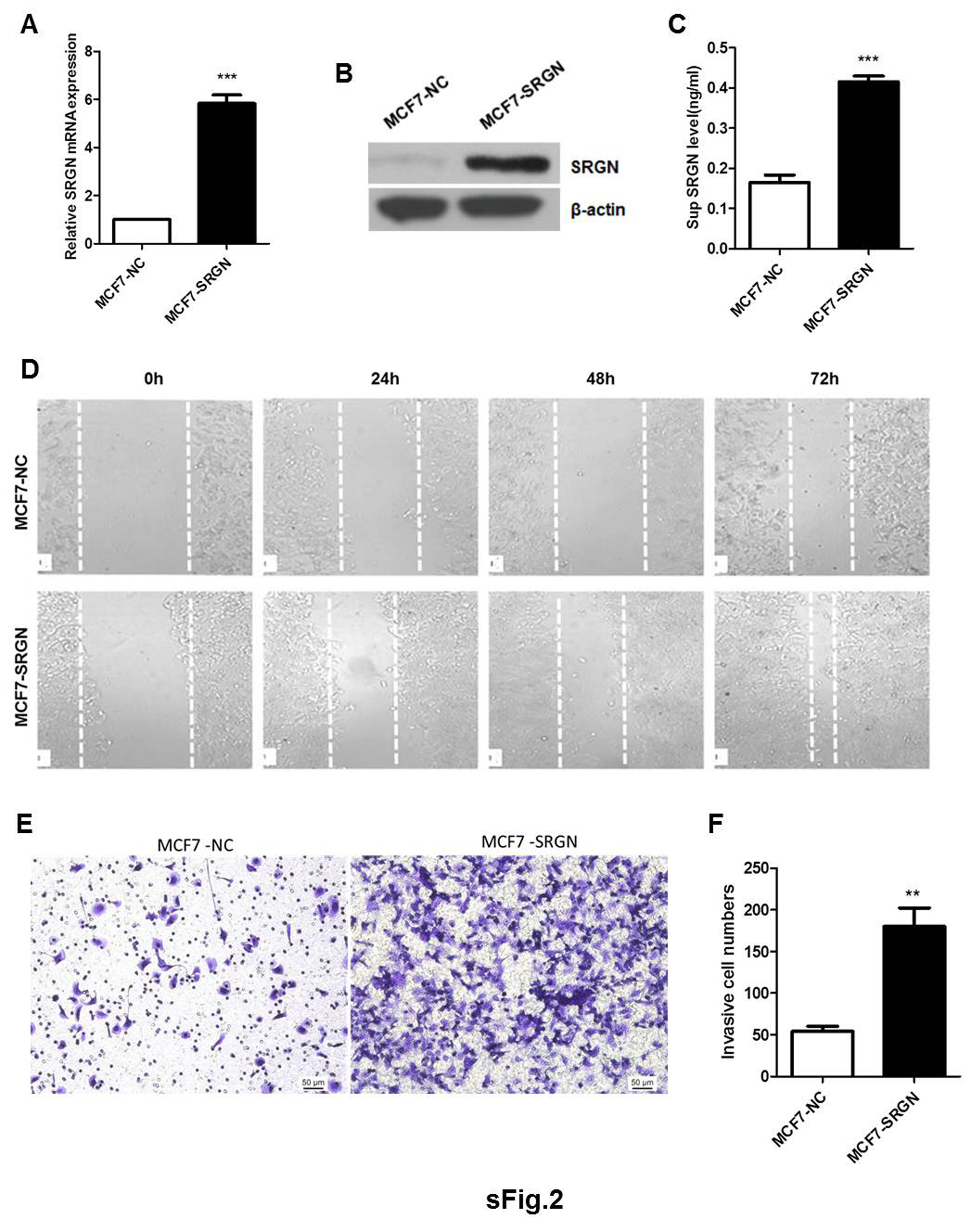

Supplement: Supplementary Figure 2 [file oncsis201753x2.tif]

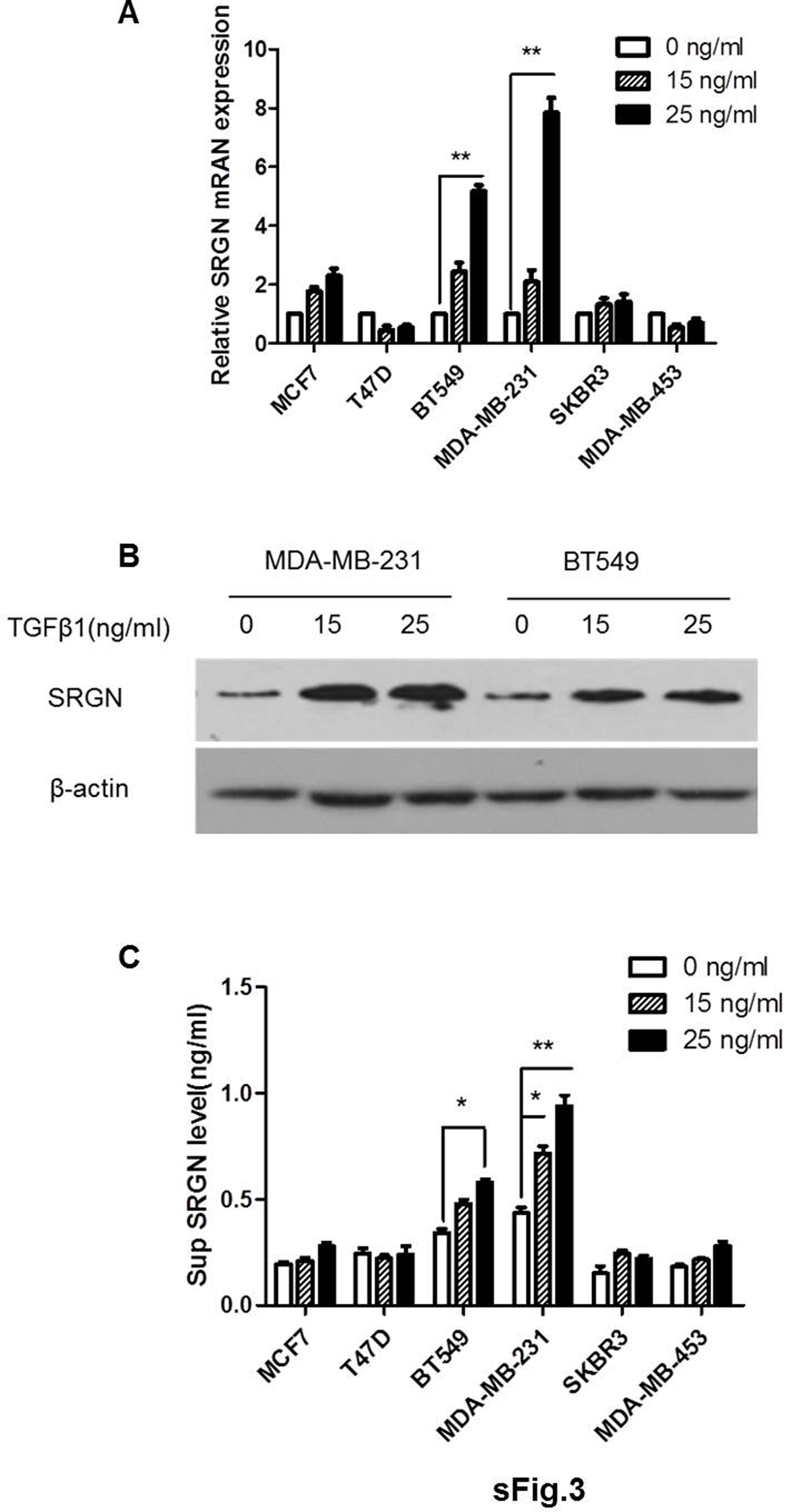

Supplement: Supplementary Figure 3 [file oncsis201753x3.tif]
